# COPB2 drives gastric cancer progression via PI3K/AKT/NF-κB signaling: a multi-omics and functional study

**DOI:** 10.1080/19336918.2026.2620945

**Published:** 2026-01-31

**Authors:** Hailong Li, Dong Wei, Xiaqing Gao, Rong Su, Chunting Yang, Ping Tang, Xiqiu Yu, Yuhong Wu

**Affiliations:** a Department of Geriatrics, Shenzhen Hospital of Integrated Traditional Chinese and Western Medicine, Shenzhen, Guangdong Province, P. R. China; b NHC Key Laboratory of Diagnosis and Therapy of Gastrointestinal Tumor, Gansu Provincial Hospital, Lanzhou, P. R. China; c Department of Internal Medicine, First School of Clinical Medicine, Gansu University of Chinese Medicine, Lanzhou, P. R. China; d Department of Preventive Medicine, Luohu District Traditional Chinese Medicine Hospital, Shenzhen, P. R. China; e Department of General Practice, Luohu Clinical College, School of Medicine, Shantou University, Shenzhen, P. R. China; f Department of Gastroenterology, Luohu District People’s Hospital, Shenzhen, P. R. China; g Department of Chinese Medicine, South China Hospital, Health Science Center, Shenzhen University, Shenzhen, P. R. China

**Keywords:** COPB2, gastric cancer, Ingenuity Pathway Analysis, microarray, tissue microarray

## Abstract

This study investigated the role of COPB2 in gastric cancer (GC) pathogenesis. Analysis of TCGA datasets and tissue microarrays revealed its upregulation in GC tissues compared to normal adjacent tissues, which was correlated with advanced tumor stage and lymphatic invasion and demonstrated significant diagnostic value (AUC = 0.895 and 0.851). Functional assays using lentiviral-mediated silencing in GC cells showed that COPB2 knockdown suppressed cell proliferation and migration, induced G0/G1-phase arrest, and promoted apoptosis. Mechanistic investigations through microarray, KEGG, and IPA analyses indicated that COPB2 dysregulation inactivated the PI3K/AKT and NF-κB signaling pathways. This led to the downregulation of key oncogenic effectors including Slug, FN1, CDH2, F2RL1, CDK6, CCND1, MMP9, CDKN2A, and SQSTM1, while upregulating tumor suppressors CDKN1B, CDKN1A, and DDIT3. In conclusion, COPB2 acts as an oncogene in GC, driving tumor progression through modulation of the cell cycle and key signaling pathways, highlighting its potential as a therapeutic target.

## GRAPHICAL ABSTRACT

**Figure:**
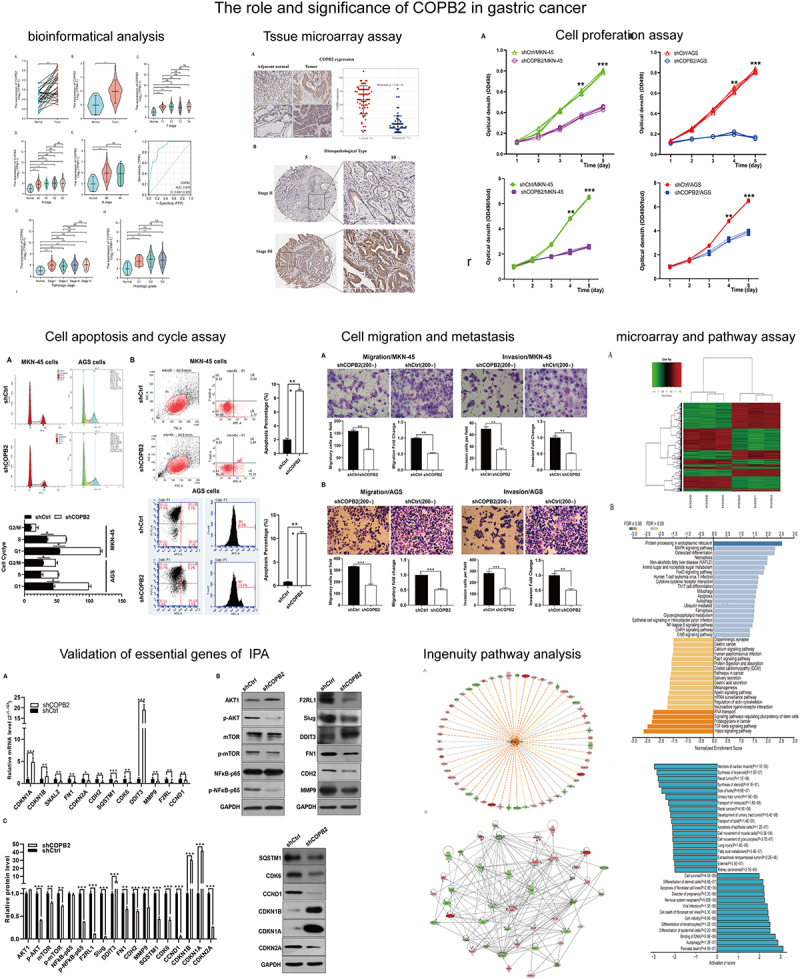

## Introduction

Gastric cancer (GC) is a common and lethal cancer worldwide with dramatic morbidity and mortality. Recent epidemiology data have revealed that the greatest incidence of gastrointestinal malignancies. of GC has increased significantly in East Asia [1]. GC continues to rank fourth in the world in terms of cancer-related death and is the fifth most common malignant cancer worldwide. The worldwide burden of this cancer is predicted to rise by 62% by 2040, despite decreasing incidence rates [2,3]. The overall prognosis of patients with GC is still poor since distant and local metastases always occur after diagnosis. Therefore, it is crucial to identify new targets for effective treatment and biomarkers for early detection. COPI coat complex subunit beta 2, abbreviated as COPB2, is a subunit of the coatomer protein complex. COPI coat complex subunit beta 2, abbreviated as COPB2, is a subunit of the coatomer protein complex that binds dilysine motifs and participates in Golgi nonclathrin-coated vesicle transportation in exocytic membrane trafficking and endocytic recycling of surface receptors [4]. Recent reports have shown that COPB2 is overexpressed in multiple cancers, such as lung adenocarcinoma [5], breast cancer [6], colorectal cancer [7], prostate cancer [8,9] and GC [10,11], and acts as a target for tumor gene silencing. Silencing COPB2 inhibits cell proliferation [9–11], arrests the cell cycle at the G1 or G2 phase [9], induces apoptosis [10], and significantly inhibits the invasion of lung cancer cells [12]. Previous studies have shown that COPB2 silencing inhibits cell proliferation and induces apoptosis [10,11]; however, whether and how COPB2 shRNA inhibits metastasis, the cell cycle of GC cells, and relevant mechanisms still need to be investigated. Therefore, we detected the effects of COPB2 silencing on migration, invasion and the cell cycle, as well as the underlying molecular mechanisms, via mRNA microarray analysis and IPA to determine the role of COPB2 in GC tumorigenesis. To be confident, this work will increase the possibility of COPB2 as a new promising potential biomarker and target therapy gene.

## Materials and methods

### Analysis of the clinical features of COPB2 via bioinformatics methods

To fully understand the significance of COPB2 in GC, we collected clinicopathological feature data via a bioinformatical tool, the Xiantao Web server (https://www.xiantao.love/) [13] (supported by the TCGA database), to analyze the relative expression levels of COPB2 in GC tissues via adjacent tissues and the significance of pathological features, such as TNM stage, histological stage, and ROC diagnostic value, to determine its potential value.

### Tissue microarrays and cell lines

The tissue microarrays (TMAs) were approved by its ethics committee of Shanghai Outdo Biotech Company with the permit number SHYJS-CP-1701010 (YBM-0–02) including 74 instances of neighboring normal tissues and 74 instances of tumor tissues of paraffin-embedded primary GC tissues for human GC-clinical specimens provided by Shanghai Outdo Biotech Company (Shanghai, China). The clinical diagnosis and demographic data, which included age, sex, tumor size, clinical stage, and tumor/node/metastasis (TNM) stage, were obtained from a company and are compiled in Table 1.

**Table 1.** Clinical characteristics of the expression of COPB2 in GC.

	Variables	low	high	total	p value	r value
sex					1	0.02
	Male	5	9	14		
	Female	20	40	60		
age					0.624	0.086
	≤64	14	23	37		
	> 64	11	26	37		
Tumor_size					0.452	0.098
	≤5 cm	16	26	42		
	> 5 cm	8	20	28		
T					0.125	−0.192
	I-III	13	35	48		
	Ⅳ	12	14	26		
N					0.12	−0.199
	N0	2	12	14		
	N1/N2/N3	23	37	60		
M					0.217	−0.16
	M0	21	46	67		
	M1	4	3	7		
Grade					0.02	−0.286
	II	4	22	26		
	III	21	27	48		
TNM					0.306	−0.129
	I-II	6	18	24		
	III-Ⅳ	19	31	50		

Note: The difference in COPB2 expression levels between Grade II and Grade III disease was statistically significant (*p* < .05).

The gastric cancer cell lines MKN-45 and AGS were purchased from the Type Culture Collection of the Cancer Institute and Hospital, Chinese Academy of Medical Sciences (CAMS) (Beijing, China). The cells were maintained in a medium composed of DMEM supplemented with 100 IU/mL penicillin and 100 μg/mL streptomycin and 10% heat-inactivated fetal bovine serum in a cell incubator with a humidified atmosphere of 5% CO_2_ at 37°C. Once the cells reached the exponential growth stage, they were collected for further experiments.

### Immunohistochemical analysis via tissue microarrays

The COPB2 protein expression in the samples was investigated via immunohistochemistry on TMA slides. To recover the antigens, the tissues were deparaffinized, rehydrated, and treated with citric acid buffer (pH 6.0) at 95–100°C for 10 min. Endogenous peroxidase activity decreased when the mixture was incubated in 3% H_2_O_2_ for longer than 10 min. Primary polyclonal anti-COPB2 antibody (1:1000; GTX65917, USA) was applied to the tissues overnight at 4°C in a humidified environment. The slides were treated with a secondary antibody (K8002, Dako, USA) for 45 min after being washed the next day with phosphate-buffered saline (PBS). A nonbiotin horseradish peroxidase detection system and DAB substrate (Dako, USA) were used for detection after washing. Hematoxylin was used as a counterstain for the tissues. A semiquantitative immunohistochemical score (0–12 points) based on the intensity (0–3 points) and proportion (0–4 points) of the immunohistochemistry data was used to assess the expression of COPB2. The staining intensity was graded as follows: 0 represented negative staining (-), 0.5 represented mild staining (0.5+), 1 represented weak staining (1+), 2 represented moderate staining (2+), and 3 represented strong staining (3+). The percentage of positive staining was ranked as follows: > 75% was ranked 4, 51–75% was ranked 3, and no staining was ranked 0. The intensity score and the amount of positive cell staining were multiplied to determine the final staining score, which ranged from 0 to 12 points.

### Lentiviral infection of gastric cancer cells

*The* GC cell lines MKN-45 and AGS were seeded in six-well plates at 5 × 10^4^ cells/well and incubated at 37°C at 5 × 10^4^ cells/well until 30% confluence was reached under an inverted microscope (IX71; Olympus Corporation, Co., Ltd., Japan). The cells were infected with negative control lentivirus (shCtrl, which was transfected with empty green fluorescent protein (GFP) lentivirus; Genechem Co., Ltd., Shanghai, China) or shCOPB2 (shCOPB2, which was transfected with shCOPB2 GFP lentivirus; Genechem Co., Ltd., Shanghai, China). A suitable volume of lentivirus was used to infect MKN-45 cells on the basis of the multiplicity of infection (MOI) determined previously [11]. The cells were cultured in medium, and the medium was replaced with fresh medium repeatedly every 12 hours. GFP-tagged fluorescence was observed under a microscope at 3 d after transduction, and cells with infection efficiencies greater than 80% were chosen for further analysis. The cells were harvested 48 hours post-transfection for further investigation.

### Silencing effect detection by RT‒qPCR analysis

The mRNA and protein levels of COPB2 were measured via RT‒qPCR and western blot, respectively, to assess the efficacy of COPB2 silencing in MKN-45 and AGS cells. In brief, the Prime ScriptTM RT Reagent Kit (Takara Bio, Dalian, China) was used to synthesize first-strand cDNA after total RNA from all the cells was extracted via the RNAiso Plus reagent (Takara Bio, Dalian, China). Next, RT‒qPCR was carried out via SYBR Master Mix (Yeasen, Shanghai, China) in a Bio-Rad CFX96 system. The real-time PCR system was operated with the following protocol: 40 cycles of 95°C for 10 s and 60°C for 30 s, followed by denaturation at 95°C for 10 min. The primer sequences (Takara Bio, Dalian, China) are provided in Table 2. When all the PCR cycles were completed, the melting curve was measured to analyze the specificity of the PCR product. The level of COPB2 mRNA was normalized to that of GAPDH mRNA via the 2^−ΔΔCt^ method. The experiment was run in triplicate and repeated at least once.

**Table 2.** Sequences of primers used in the study.

Gene	primer	Sequence (5’−3’)	Product size (bp)
COPB2	Forward Rerverse	GTGCTCTCAAGCCGGTAGG GTGGGGACAAGCCATACCTC	211
CDKN1A	Forward	TCAAATCGTCCAGCGACCTTC	125
	Rerverse	CATGCCCTGTCCATAGCCTCTAC	
CDKN1B	Forward	CAAATGCCGGTTCTGTGGAG	177
	Rerverse	TCCATTCCATGAAGTCAGCGATA	
CCND1	Forward	ACCAGCTCCTGTGCTGCGAAGTG	157
	Rerverse	GACGGCAGGACCTCCTTCTGCACA	
SNAIL2	Forward	TTTCCAGACCCTGGTTGCTTC	102
	Rerverse	CTCAGATTTGACCTGTCTGCAAATG	
CDK6	Forward	GTGACCAGCAGCGGACAAATAA	119
	Rerverse	AGCAAGACTTCGGGTGCTCTGTA	
DDIT3	Forward	ATCTGCACCAAGCATGAACAA	115
	Rerverse	AGGGTCACATCATTGGCACTA	
F2RL1	Forward	ACAGACACGTCCTCATAACATTAAACA	151
	Rerverse	TCCCTCACCTCAAAGAAACACTCC	
SQSTM1	Forward	CATCGGAGGATCCGAGTGTG	114
	Rerverse	TTCTTTTCCCTCCGTGCTCC	
MMP9	Forward	TACTGTGCCTTTGAGTCCG	162
	Rerverse	TTGTCGGCGATAAGGAAG	
FN1	Forward	ACAAACACTAATGTTAATTGCCCA	119
	Rerverse	AACTCCCAGGGTGATGCTTG	
CDH2	Forward	CGAATGGATGAAAGACCCATCC	171
	Rerverse	GCCACTGCCTTCATAGTCAAACACT	
GAPDH	Forward	AGGTCGGTGTGAACGGATTTG	123
	Rerverse	TGTAGACCATGTAGTTGAGGTCA	

### Silencing effect detection by western blot analysis

GC cells from the shCOPB2 and shCtrl groups were collected, washed twice with PBS, and lysed for 5 min in ice-cold lysis buffer (50 mM Tris-HCl, pH 7.5; 150 mM NaCl; 1% Triton X-100; 1 mM ethylene diamine tetraacetic acid; 1 mM PMSF; and 1% sodium deoxycholate). Total protein was extracted from the cell lysis buffer. The protein concentration was determined via a Bio-Rad protein assay kit (Bio-Rad Laboratories, Shanghai, China). Protein samples were separated via 10% SDS‒PAGE and then transferred to PVDF membranes. Following the recommended protocol, the blots were incubated at room temperature with primary antibody (mouse anti-Flag; Sigma, 1:2000; China headquarters, Shanghai, China). The corresponding horseradish peroxidase (HRP)-conjugated secondary antibody (goat anti-mouse IgG, 1:2000, Santa Cruz Biotechnology, Dallas, Texas, USA) was incubated with the blots for 1.5 hours after they were washed in 5% nonfat milk in TBST (containing Tris-HCl, NaCl, and Tween-20) saline at room temperature. Chemiluminescence was used (Thermo Scientific Pierce, Shanghai, China) to identify bands, which were then scanned to obtain corresponding images. The GAPDH (Mouse Anti-Flag, 1:2000, Santa Cruz Biotechnology, Dallas, Texas, USA) housekeeping control was used as a normalization tool for the triplicate western blotting assays. For semiquantification and comparison between the two groups, the ratio of COPB2 protein expression to GAPDH expression was utilized. The final immunological reaction results were quantified via ImageJ software (NIH Image for the Macintosh, USA).

### Cell proliferation

Following transfection, the two groups of cells were trypsinized and counted. In each well of a 96-well plate, 3000 cells were plated, and the plates were then incubated at 37°C for 24, 48, 72, 96, and tentative hours. The cells were then plated starting on the second day, after which the number of cells was tracked for five days in a row. Twenty microliters of 5 μmol/L MTT (3-(4,5-dimethylthiazol-2-yl)-2,5-diphenyltetrazolium bromide) (Sigma‒Aldrich, St. Louis, MO, USA) dissolved in phosphate-buffered saline (PBS) was added to each well, and the plate was incubated at 37°C for 4 hours before the cell culture was terminated for 5 consecutive days. After the plate was centrifuged at 1000 × g for two min, the medium was extracted. Next, 150 µL of DMSO was added to each well. The absorbance was measured spectrophotometrically at 490 nm with a benchmark microtiter plate reader (Bio-Rad Laboratories, Hercules, CA, USA) following a 5-minute incubation period at 37°C.

### Quantification of apoptosis by flow cytometry

After being harvested with 0.25% trypsin, the cells were once again cleaned with 4°C ice-cold D-Hanks (pH = 7.2–7.4). After being rewashed with 1 mM binding solution and centrifuged for 3 min at 1300 rpm, the cells were centrifuged for 5 min at 1300 rpm. After being resuspended in 200 μL of binding buffer containing 10^6^ cells/mL, apoptosis was measured via an Annexin V-APC apoptosis detection kit (eBioscience, San Diego, CA, United States). After remaining at room temperature for 15 min, 100 μL of the cell suspension was treated with 10 μL of Annexin V-APC in the dark. The tagged cells were added to 400–800 μL of 1× binding buffer, depending on the quantity of cells. The proportion of apoptotic cells was examined via flow cytometry. This experiment was conducted three times. At least one repetition of the experiment was conducted in triplicate.

### Cell cycle analysis

Following successful transduction, the GC cell lines MKN-45 and AGS from the shCOPB2 and shCtrl groups were collected for univariate cell cycle assays. The assay was performed by using ethanol-fixed cells stained with propidium iodide in buffer containing RNase A (Fermentas, USA). The DNA content was assessed via flow cytometry (Guava easyCyte 6HT, Millipore, USA), and cell cycle analysis was performed via the ModFit software package (Verity Software House, San Diego, USA). This assay was also repeated in triplicate.

### In vitro cell migration and invasion assays

Following successful transduction, gastric cancer cells in the shCOPB2 and shCtrl groups were prepared for cell migration and invasion assays in vitro. A Transwell chamber assay (BD Biosciences, Franklin Lakes, NJ, USA) was performed to observe cellular invasion in vitro. Briefly, the upper chamber of the transwell was coated with 60 μL of Matrigel (BD Biosciences) diluted with serum-free medium (1:50). The cells were then seeded into the upper chamber in 200 μL of serum-free medium at a density of 4 × 10^4^ cells/chamber. The lower chamber was filled with 750 μL of medium containing 10% FBS. Following incubation at 37°C for 48 h, the cells were fixed with 4% polyoxymethylene (Shanghai Macklin Reagent Co., Ltd., Shanghai, China) for 10 min and stained with 0.5% crystal violet (Shanghai Macklin Reagent Co., Ltd.) for 30 min. The cells that did not invade through the pores of the membrane were wiped away with a cotton swab, and those that migrated and invaded the cells were counted under an inverted microscope. The experiment was also repeated three times.

### Screening aberrantly expressed genes via an Affymetrix mRNA microarray

Following transduction, MKN-45 cells in the shCOPB2 and shCtrl groups were collected. Total RNA was isolated via TRIzol reagent (Invitrogen, Carlsbad, CA, USA) according to the manufacturer’s instructions. RNA quality and quantity were measured via a NanoDrop spectrophotometer (ND-1000, Thermo Fisher Scientific, Wilmington, DE, USA), and RNA integrity was determined by gel electrophoresis. The concentration and purity of total RNA were assessed by A_260_ and A_280_ using an ultraviolet spectrophotometer. Only the RNA samples with A260/A280 ratios > 1.8 were used in this study. The commercially available PrimeView™ Human Gene Expression Array experiment was performed by Genechem Corporation (Co., Shanghai, China). The labeling, hybridization, washing, and scanning procedures were performed according to the standard operating procedures provided by Affymetrix. Briefly, total RNA was used to synthesize cDNA via an in vitro transcription reaction. The cDNA was labeled with biotin and T7 Enzyme Mix. After hybridization, nonspecifically bound molecules were removed from the microarray with two wash buffers. The arrays were subsequently scanned with a GeneChip® Scanner 3000 (Affymetrix, Cleveland, USA), and the hybridization data were analyzed via the Gene Matrix cloud service (http://gcloud.taogene.com).

### RT‒qPCR and western blotting validation of aberrantly expressed genes

Total RNA and protein were extracted from GC cells in the shCOPB2 and shCtrl groups. All procedures of RNA extraction, reverse transcription and amplification were the same as the procedures mentioned above. The primer sequences (Takara Bio, Dalian, China) are provided in Table 2. Similarly, all procedures for protein isolation, gel electrophoresis, transfer to PVDF membranes, and immunoblotting were the same as the procedures described above. The blots were incubated with the appropriate primary antibodies against Slug (1:1000, #9585, CST), FN1 (1:1000, YC0013, Immunoway), CDH2 (1:1000, ab18203, ABCAM), F2RL1 (1:1000, #6976, CST), CDK6 (1:1000, #3136, CST), MMP9 (1:1000, #13667, CST), CCND1 (1:500, #2978, CST), CDKN2A (1:1000, ab108349, ABCAM), CDKN1B (1:1000, #3686, CST), CDKN1A (1:1000, #2947, CST), SQSTM1 (1:1000, #88588, CST) and DDIT3 (1:1000, ab11419, ABCAM) at room temperature. After being washed with 5% nonfat milk in TBST saline (20 mM Tris – HCl, pH 7.4, 137 mM NaCl, and 0. 1% Tween-20) at RT for 1 h. In addition, to explore the possible signaling pathways involved in the effects induced by COPB2 silencing, nonphosphorylated proteins such as AKT1 (1:1000, ab124341, ABCAM), mTOR (1:1000, #2983, CST), NFκB-p65 (1:1000, ab31481, ABCAM), and phosphorylated proteins such as p-AKT (1:1000, #4060, CST), p-mTOR (1:1000, ab109268, ABCAM), and p-NFκB-p65 (1:1000, 3033S, CST) were also detected via western blotting. The experiments were performed in triplicate, with GAPDH (1:1000, sc-32233, Santa Cruz) used as a housekeeping control for normalization. The membranes were further incubated with the corresponding horseradish peroxidase (HRP)-conjugated secondary goat anti-rabbit IgG or goat anti-mouse IgG (1:2000; sc-2004 or sc-2005, Santa Cruz) for 90 min. The experiments were repeated three times, and the target gene-to-GAPDH ratio was calculated for semiquantification and comparison between the two groups.

### Statistical analysis

The Wilcoxon method and paired sample test were used to compare the expression of COPB2 in the nonpaired and paired samples of TCGA data respectively. The Welch one-way ANOVA test and multiple hypothesis test (Games Howell post hoc test or Tukey HSD post hoc test) were used to analyze the clinical features of COPB2 expression of TCGA data. The measurement of experimental data is expressed as the means ± standard deviations from at least 3 separate experiments and were evaluated via Student’s two-tailed t test. The Wilcoxon method was used to compare the expression of COPB2 in the clinical samples of the TMA, the Fisher’s test data was used to compare of the clinical features of the TMA, and the *r* value was the correlation coefficient. Differences with a *p* value of < 0.05 were considered statistically significant.

## Results

### The overexpression of COPB2 is closely related to pathological features

**Figure 1.**
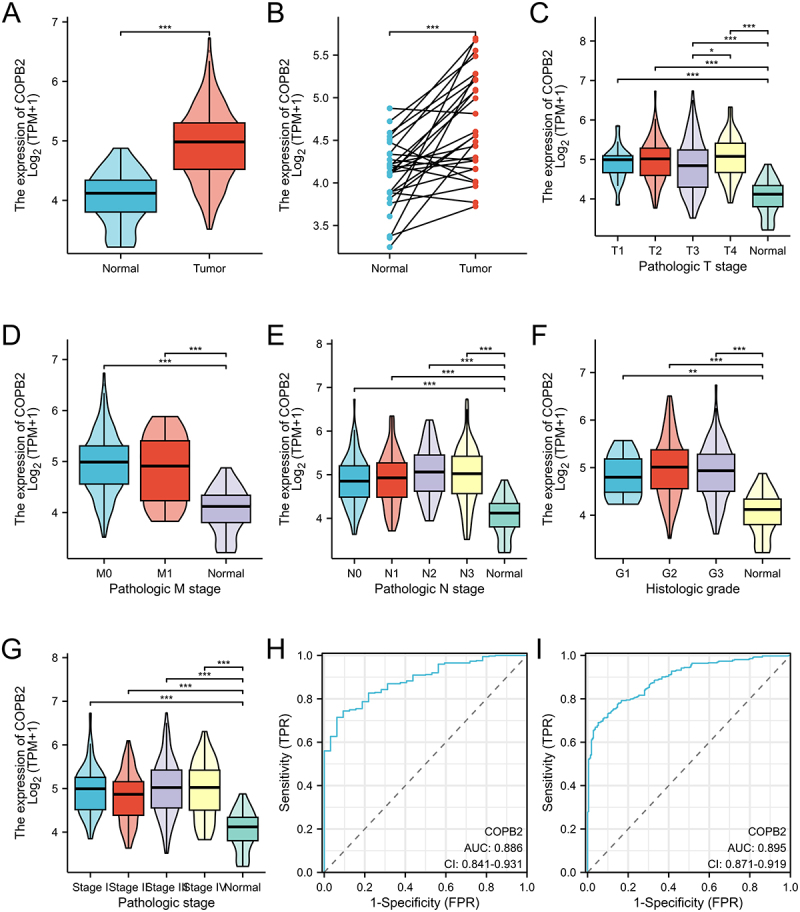
(A, B, C, D, E, F, G, H, I). The overexpression of COPB2 is closely related to pathological features.

COPB2 is elevated in GC tissues compared with surrounding normal tissues, according to a bioinformatics study (Figure 1(A,B)). The histological grades 1, 2, and 3 of the GC tissues presented higher levels of COPB2 than did the normal tissues (Figure 1C); the T1, T2, T3, and T4 stages of the GC tissues presented higher levels of COPB2 than did the normal tissues did (Figure 1D); the N0, N1, N2, and N3 stages of the GC tissues presented higher levels of COPB2 than did the normal tissues did (Figure 1E); and the M0 and M1 stages of the GC tissues presented higher levels of COPB2 than did the normal tissues did (Figure 1F). Compared with that in normal tissues, COPB2 is upregulated in GC tissues of pathological stages I, II, III, and Ⅳ (Figure 1G). Moreover, ROC analysis revealed that the AUC square of COPB2 overexpression equals 0.895 (95% CI 0.871–0.919) in the TCGA-STAD data containing 407 samples (Figure 1H) and 0.851 (95% CI 0.793–0.908) (Figure 1I) in the TCGA_GTEx-STAD of XENA containing 624 samples data, which is a valuable diagnostic indicator of GC. In summary, pathological aspects are linked to the overexpression of COPB2, and further research is needed to determine the growth of tumors, metastasis ability, early diagnostic value, and prognostic value of COPB2 in GC.

Bioinformatic analysis revealed that COPB2 is upregulated in gastric cancer tissues compared with adjacent normal tissues (Figure 1(A,B)). COPB2 is also upregulated in gastric cancer tissues from the T1, T2, T3, and T4 stages compared with normal tissues (Figure 1C); COPB2 is upregulated in gastric cancer tissues from the N0, N1, N2, and N3 stages compared with normal tissues (Figure 1D); and COPB2 is upregulated in gastric cancer tissues from the M0 and M1 stages compared with normal tissues (Figure 1E). COPB2 is upregulated in gastric cancer tissues of histological grade 1, grade 2, and grade 3 compared with normal tissues (Figure 1F). COPB2 is upregulated in gastric cancer tissues of pathological stages I, stage II, stage III, and stage Ⅳ compared with normal tissues (Figure 1G). Additionally, ROC analysis revealed that the AUC square of the overexpression of COPB2 equals equals 0.895 (95% CI 0.871–0.919) in the TCGA-STAD data containing 407 samples (Figure 1H) and 0.851 (95% CI 0.793–0.908) (Figure 1I) in the TCGA_GTEx-STAD of XENA containing 624 samples data, which is a diagnostic sign of gastric cancer. In summary, the upregulation of COPB2 is associated with pathological features, and in-depth studies on tumor growth, metastasis, and early diagnostic value in gastric cancer are needed. *p < .05, **p < .01, ***p < .001, compared with the normal group.

### COPB2 overexpression is directly related to pathologic features in GC

**Figure 2.**
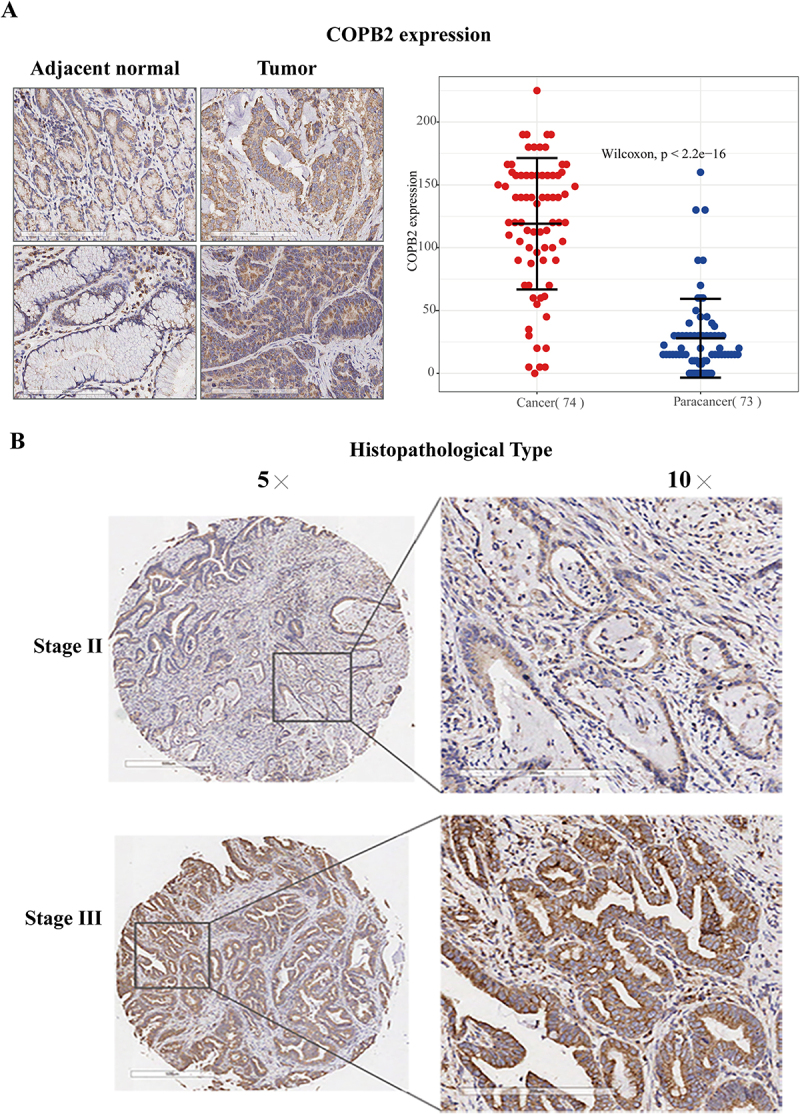
(A, B). The overexpression of COPB2 is directly related to the pathologic features of GC.

By examining COPB2 in GC tissues, it will be possible to ascertain the clinical importance of COPB2 in the emergence and development of GC. This study employed a tissue microarray, which included 74 tumor tissues (grades I – IV) and 74 normal tissues, to detect and compare the expression of COPB2 by immunohistochemical analysis. Immunohistochemical labeling revealed that the majority of the COPB2 protein was expressed in the GC cell nucleus (Figure 2A). Furthermore, there was a statistically significant increase in COPB2 expression in tumor tissues compared with surrounding normal tissues (*p* < .05, Figure 2B). In this group, the percentage of COPB2-positive samples exhibited a comparable pattern (*p* < .05, Table 1). The results of Fisher’s test revealed a significant correlation between the elevated expression of COPB2 and clinical grade (*p* = .02). With respect to T, N, and M stage (*p* = .12, *p* = .125, and *p* = .217, respectively), sex (*p* = 1), age (*p* = .623), and tumor size (*p* = .542), no link was found between the expression of COPB2 and the TNM stage classification (*p* > .05, Table 1). Additionally, an analysis of the relationship between COPB2 expression and the clinical phases of GC was conducted. The findings indicated that the difference in COPB2 expression levels between Grade II and Grade III disease was statistically significant (*p* < .05, Table 1). These findings suggest that COPB2 might be used to predict a patient’s clinicopathological features.

This TMA, which included 74 tumor tissues (grades I – IV) and 74 normal tissues, is appropriate for evaluating the expression of COPB2 in GC tissues via immunohistochemistry. The results revealed that the COPB2 protein was expressed mostly in the nucleus of GC cells (Figure 2A). Furthermore, there was a statistically significant increase in COPB2 expression in tumor tissues compared with surrounding normal tissues (*p* < .05, Figure 2B). In this group, the percentage of COPB2-positive samples exhibited a comparable pattern (*p* < .05, Table 1). The statistical results revealed a significant correlation between elevated COPB2 expression and clinical grade (*p* = .02). With respect to T, N, and M stage (*p* = .12, *p* = .125, and *p* = .217, respectively), sex (*p* = 1), age (*p* = .623), and tumor size (*p* = .542), there was no link between the expression of COPB2 and TNM stage (*p* > .05, Table 3).

**Table 3.** A total of 42 genes related to disease and function were identified via IPA.

Diseases or Functions Annotation	Autophagy of cells	Perinatal death	Autophagy	Binding of DNA	Differentiation of epidermal cells	Differentiation of keratinocytes	Cell viability	Cell death of fibroblast lines	Viral Infection	Nervous system neoplasm	Disorder of pregnancy	Apoptosis of fibroblast lines	Differentiation of dermal cells	Cell survival	Kidney carcinoma	Edema	Extraadrenal retroperitoneal tumor	Fatty acid metabolism	Lung injury	Cell movement of granulocytes	Cell movement of muscle cells	Apoptosis of epithelial cells	Transport of lipid	Development of urinary tract tumor	Renal cancer	Transport of molecule	Urinary tract tumor	Size of body	Synthesis of steroid	Renal tumor	Synthesis of terpenoid	Necrosis of cardiac muscle
ANXA1					√	√			√	√			√				√	√							√	√						
CAV1				√			√		√	√				√	√	√	√	√	√				√		√	√	√	√		√	√	
CCND1	√	√	√	√	√	√	√	√		√			√	√	√	√	√				√	√		√		√	√	√		√		
CDH1					√	√			√	√			√	√	√		√					√		√	√		√			√		
CDH5							√							√																		
CDK6		√					√	√		√				√			√				√	√						√				
COPB1									√																							
COPB2									√					√																		
DDIT3	√		√	√			√	√	√			√		√										√								
EDN1		√		√			√							√	√	√	√	√	√	√		√			√	√	√		√	√	√	√
EGR1				√			√	√	√			√		√			√	√	√		√	√			√	√						
EIF2AK3	√		√				√	√	√	√	√	√		√			√	√	√	√							√	√				
F2R							√		√					√		√			√	√	√				√	√						
F2RL1		√					√		√					√		√			√						√	√						
FGFR3		√					√			√					√		√	√									√	√		√		
FOXO3	√		√	√			√	√	√	√		√		√		√	√	√	√					√	√	√	√			√		√
FTH1							√	√				√		√											√	√						
GADD45A							√	√				√		√								√										
ID1		√		√	√	√		√				√	√				√					√						√				
ID2		√		√			√	√		√		√		√								√						√				
ID3				√						√																						
IGFBP1										√												√						√				
IL1R1				√						√					√				√	√				√			√	√		√		
IL24			√	√	√	√	√			√			√	√																		
ITPR1	√		√					√				√														√						
JUN				√			√	√	√			√		√		√						√					√					
MCAM							√		√					√			√															
MKI67										√																	√			√		
MMP9							√		√	√	√			√	√		√			√	√			√			√	√	√	√	√	
MYO6																									√							
NOTCH3											√						√										√					
NOV							√							√			√				√											
RB1CC1	√		√				√		√	√				√		√	√					√					√					
RND3					√	√							√									√										
RNF2							√			√				√			√															
SCYL1																																
SOX4		√							√					√		√																
SP1				√														√								√			√		√	
SPRY2		√							√																							
SQSTM1	√		√				√		√					√												√	√					
SRSF3							√							√			√								√	√	√					
TIMP3							√							√	√	√						√		√				√		√		
WBP2																																

### COPB2 silencing inhibited gastric cancer cell proliferation

**Figure 3.**
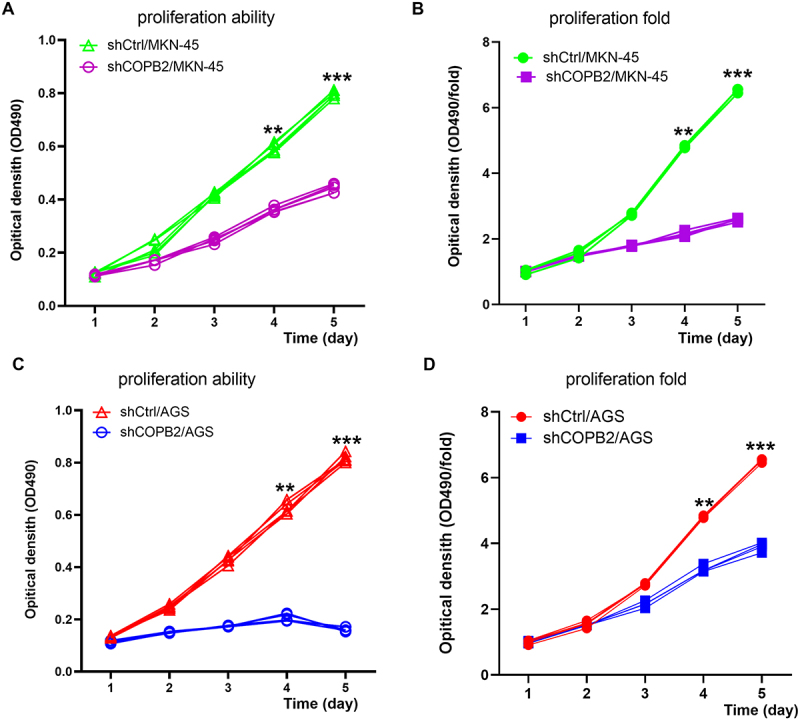
(A, B, C, D): effects of COPB2 silencing on the proliferation of gastric cancer cells, as determined by MTT detection.

The MTT results demonstrated that on the fourth and fifth days after COPB2 was significantly silenced in MKN-45 and AGS cells, the number of cells and the fold change in proliferation in the Lv-shCOPB2 group were significantly lower than those in the Lv-shCtrl group (Figure 3(A–D)). These findings suggest that COPB2 suppression might prevent cell proliferation.

The MTT assay was conducted, and the absorbance at 490 nm was detected and compared to determine the proliferation ability of each day. OD490/fold is the OD490-fold of each experimental group from day 1 to day 5 relative to day 1, which represents the proliferation fold of each day, indicating the proliferation ability of active MKN-45 and AGS cells in the Lv‑shCOPB2 and Lv‑shCtrl groups. The proliferation rate of MKN-45 and AGS cells was significantly inhibited in the Lv‑shCOPB2 infection group at 4 and 5 days post-infection (*p* < .05), indicating that the COPB2 gene may be significantly associated with the proliferation ability of MKN-45 cells, as shown in Figure 3(A,B), and AGS cells, as shown in Figure 3(C,D). *p < .05, compared with the Lv‑shCtrl group. ***p < .01, compared with the Lv‑shCtrl group.

### COPB2 silencing induced cell cycle arrest at the G0/G1 stage and apoptosis

**Figure 4.**
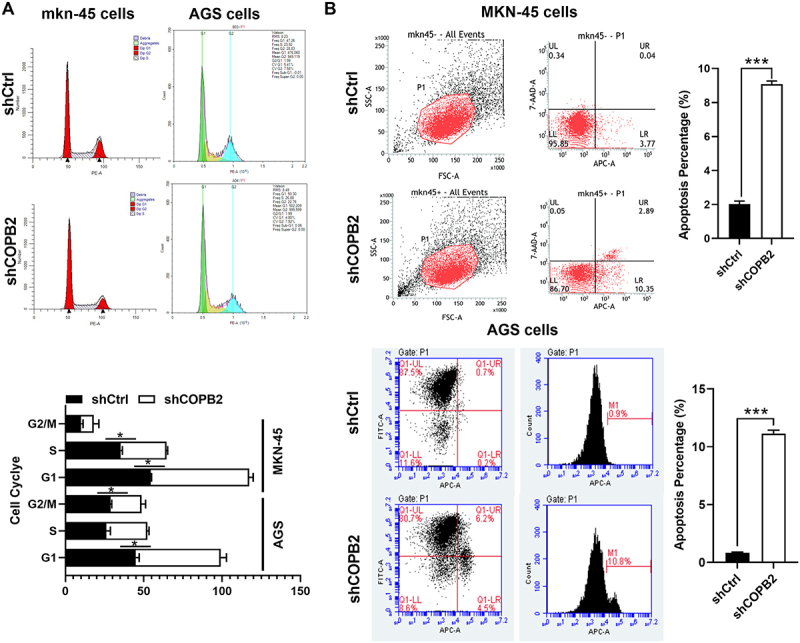
(A, B): effects of COPB2 silencing on apoptosis and the cell cycle, as determined by FCW detection.

To determine whether COPB2 silencing affects the cell cycle in MKN-45 and AGS cells, a cell cycle test was performed to compare COPB2-silenced MKN-45 and AGS cells to shCtrl cells. After 3 days of culture, more cells in the G0/G1 phase and fewer cells in the S phase were in the shCOPB2 group than in the shCtrl group (*p* < .05, Figure 4A). However, there was no significant difference in G2/M phase cells between the shCOPB2 and shCtrl groups, indicating that silencing COPB2 could arrest the cell cycle at the G0/G1 phase. The detection of the percentage of apoptotic cells revealed that the proportion of apoptotic cells was significantly greater in COPB2-silenced cells than in control cells (Figure 4(B)). These data suggested that COPB2 silencing could induce apoptosis.

After infection with the shRNA lentivirus and culture for 5 days, the number of apoptotic cells in the Lv-sh COPB2 group was significantly greater than that in the Lv-shCtrl group (*p* < .001, Figure 4(A,B)), suggesting that the COPB2 gene was significantly correlated with the apoptosis of gastric cancer cells. Compared with those in the shCtrl group, the number of cells in the G0/G1 phase in the shCOPB2 group increased, and the number of cells in the S and G2 phases in the shCOPB2 group decreased (*p* < .05, Figure 4(A,B)). These findings indicate that COPB2 silencing could arrest the cell cycle at the G0/G1 phase. *p < .05, compared with the Lv‑shCtrl group. ***p < .001, compared with the Lv‑shCtrl group.

### COPB2 silencing inhibited gastric cancer cell invasion and migration in vitro

**Figure 5.**
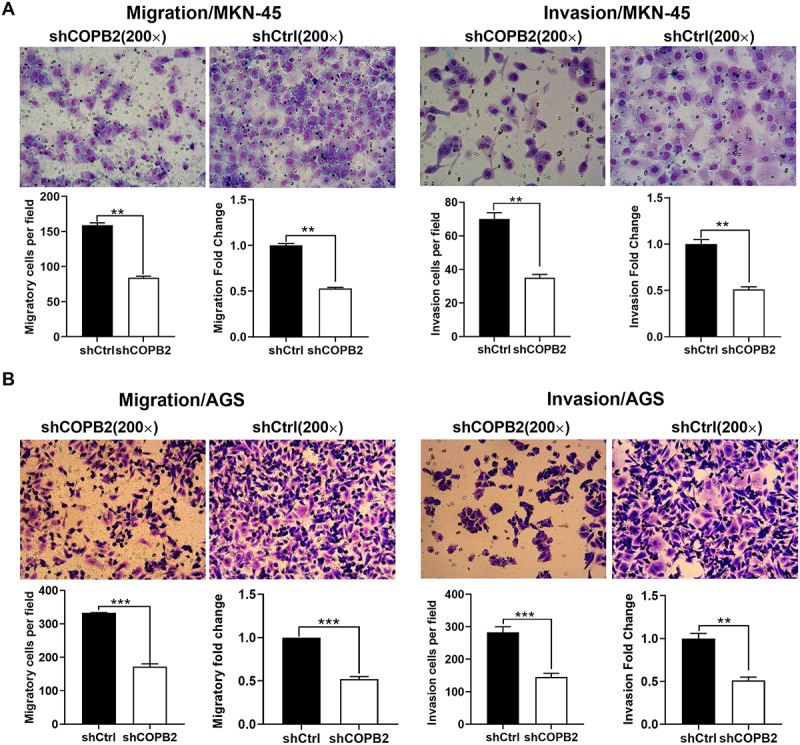
(A, B): COPB2 silencing inhibits gastric cancer cell invasion and migration in vitro.

The present study explored whether COPB2 silencing could inhibit GC cell migration and invasion by performing invasion and migration assays in a transwell chamber. The results of the transwell chamber experiments revealed that COPB2 silencing considerably reduced the capacity of MKN-45 and AGS cells to migrate and invade compared with that of cells in the shCtrl group (*p* < .05, Figure 5(A, B)). These findings suggest that in vitro GC cell migration and invasion are inhibited by COPB2 silencing.

The present study determined whether COPB2 overexpression inhibits gastric cancer cell migration and invasion. Gastric cancer cell motility was assessed via a wound-healing assay. Transwell chamber assays demonstrated that COPB2 silencing significantly inhibited the invasion and migration capacity of MKN-45 cells (*p* < .05, Figure 5A) and AGS cells (*p* < .05, Figure 5B) compared with those of shCtrl-treated cells. These results indicate that COPB2 silencing could inhibit gastric cancer cell invasion and migration in vitro. **p < .01, compared with the Lv‑shCtrl group. ***p < .001, compared with the Lv‑shCtrl group.

### Affymetrix mRNA microarray analysis of aberrantly expressed genes

**Figure 6.**
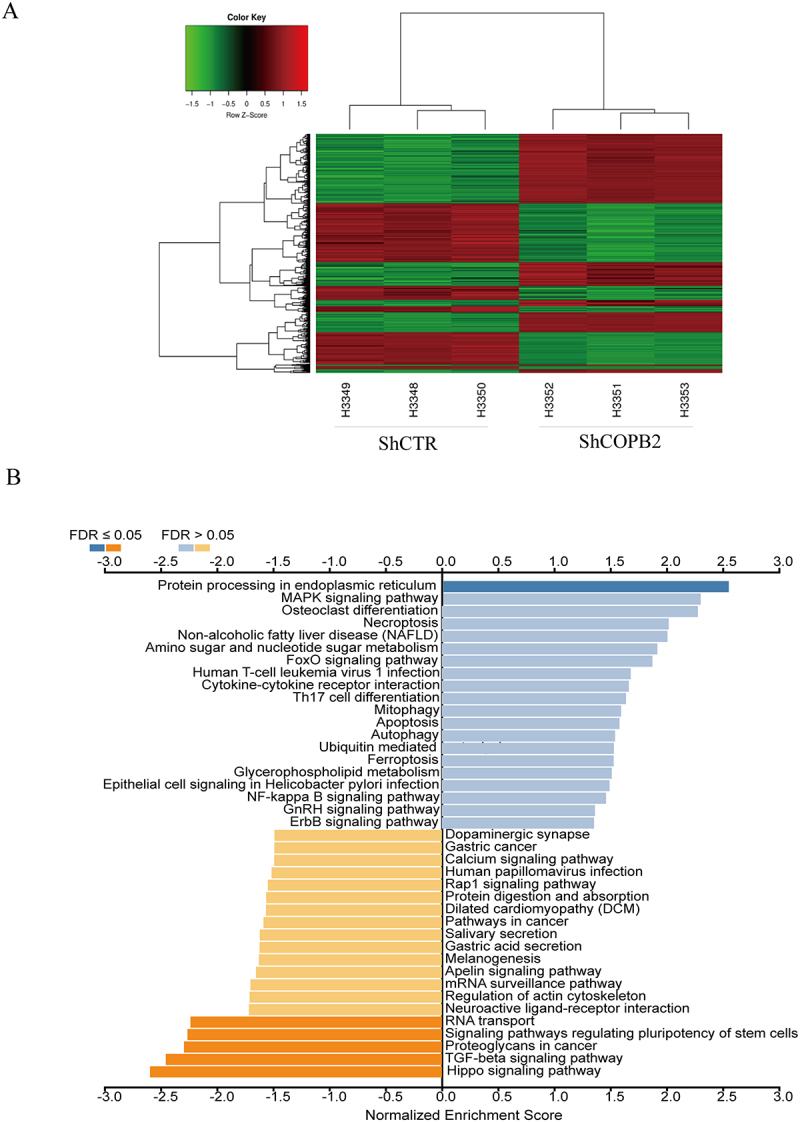
(A, B): Affymetrix mRNA microarray assay and KEGG pathway enrichment analysis.

By comparing the aberrantly expressed genes between the Lv-shCtrl group and the Lv-shCOPB2 group of MKN-45 cells, an Affymetrix mRNA microarray was used to screen and analyze the regulatory mechanism of COPB2 in the carcinogenesis of GC. In the Lv-shCOPB2 group of cells and the Lv-shCtrl group of cells, COPB2 silencing resulted in 1211 differentially expressed genes (DEGs), of which 632 were considerably upregulated and 579 were downregulated (cutoff ≥1.5, Figure 6(A)).

Microarray assays revealed that 1211 genes, including 632 upregulated genes and 579 downregulated genes, were aberrantly expressed between MKN-45 cells in the Lv-shCOPB2 group and MKN-45 cells in the Lv-shCtrl group (cutoff ≥1.5, Figure 6A). The 1211 screened DEGs were uploaded to the WebGestalt tool (http://www.webgestalt.org/) for analysis of relevant KEGG pathways. The findings demonstrated that the DEGs were enriched in pathways associated with carcinogenesis, including those linked to NF-kappa B signaling, TNF signaling, autophagy control, FoxO and HIF-1 signaling, TGF-beta signaling, proteoglycans in cancer, and others (Figure 6B).

### KEGG pathway analysis of DEGs

KEGG pathway enrichment analysis was carried out in the following steps to investigate the possible mechanisms of aberrantly expressed genes related to distinctive features of tumorigenesis in GC cells. First, the 1211 screened DEGs were uploaded to the WebGestalt tool (http://www.webgestalt.org/) for analysis of relevant KEGG pathways. This finding demonstrated that the DEGs were enriched in pathways associated with carcinogenesis, including those linked to NF-kappa B signaling, TNF signaling, autophagy control, FoxO and HIF-1 signaling, TGF-beta signaling, proteoglycans in cancer, and others. In summary, COPB2 silencing resulted in some DEGs being enriched in tumor-related pathways, making it easier for relevant pathway analysis to choose aberrantly expressed genes to uncover molecular mechanisms (Figure 6B).

### Ingenuity Pathway Analysis of DEGs

**Figure 7.**
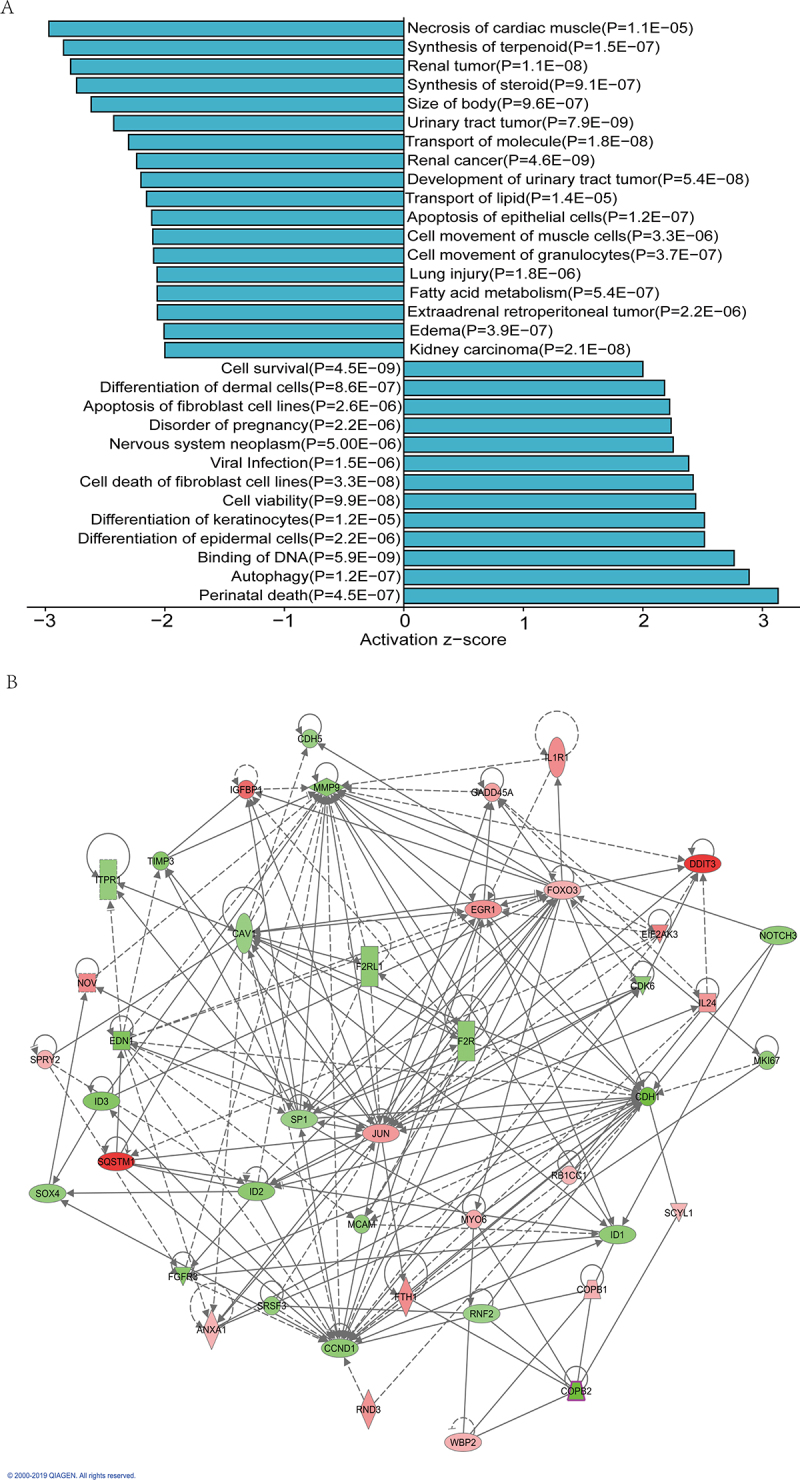
(A, B): Ingenuity Pathway Analysis of aberrantly expressed genes.

This section includes functional and illness analyses, as well as an investigation of interaction networks via IPA. The investigation of illness and function caused by COPB2 silencing via IPA revealed that kidney carcinoma, edema, extra-adrenal retroperitoneal tumors, fatty acid metabolism, lung injury, granulocyte and muscle cell motility, epithelial apoptosis, lipid transport, urinary tract tumor development, renal cancer, molecular transport, urinary tract tumors, body size, steroid synthesis, renal tumors, and synthesis were among the illnesses and functions caused by COPB2 silencing that were investigated via IPA. Terms associated with tumors, such as apoptosis, viability, death, autophagy, survival, and differentiation, are triggered. The important phrases clarified the effects of COPB2 silencing and shed light on the function and importance of COPB2 in GC cells. (Figure 7A & Table 3).

In this section, disease and functional analysis and subsequent analysis of interaction networks based on IPA were performed. The investigation of illness and function caused by COPB2 silencing via IPA revealed that tumor-related terms, such as apoptosis, viability, death, autophagy, survival, and differentiation, were activated. The significant terms described the consequences of COPB2 silencing, providing insight into the role and significance of COPB2 in gastric cancer cells (Figure 7A). Additionally, an analysis of interaction networks via IPA revealed a total of 42 aberrantly expressed genes from disease, and a functional analysis via IPA was performed. The IPA tool was used to generate an interaction map that highlights the internal interaction links. All 42 genes were implicated in relevant diseases and functions; considering the role of these genes in the interaction networks of IPA and their functions related to the cell cycle, metastasis, and important pathways, they were examined to identify genes that need to be further validated (Figure 7B).

Additionally, an analysis of interaction networks via IPA revealed a total of 42 aberrantly expressed genes from disease, and a functional analysis via IPA was performed. The IPA tool was used to generate an interaction map that highlights the internal interaction links. All 42 genes were implicated in relevant diseases and functions of IPA; these genes related to the cell cycle, metastasis, and important pathways were examined to identify which genes need further validation (Figure 7B & Table 3).

### Screened aberrantly expressed genes were experimentally validated

**Figure 8.**
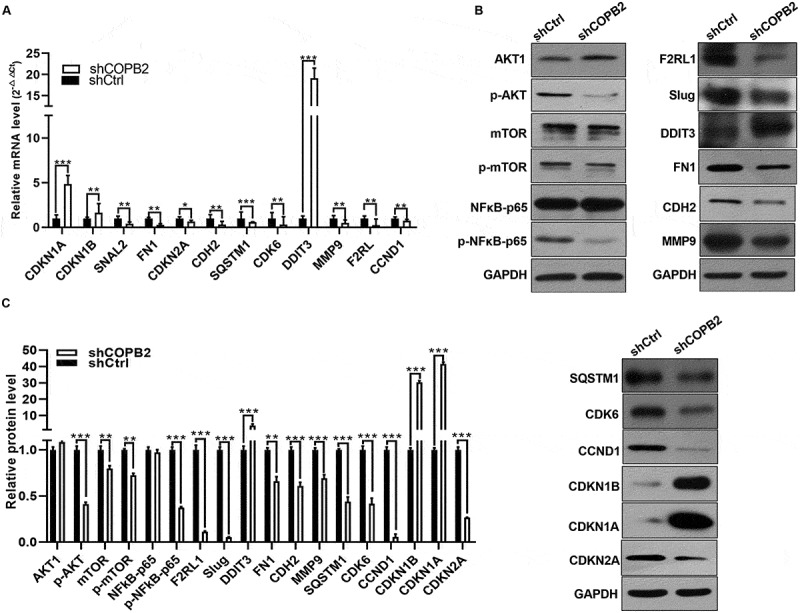
(A, B, C): effects of COPB2 silencing on aberrantly expressed genes validated by RT‒qPCR and western blotting.

To delve deeper into the molecular mechanisms at play, a subset of DEGs among 42 genes of IPA associated with autophagy, survival, differentiation, and viability was chosen for validation. The validation experiment revealed that several genes, including Slug, FN1, CDH2, F2RL1, CDK6, CCND1 MMP9, CDKN2A, and SQSTM1, are downregulated, whereas CDKN1B, CDKN1A, and DDIT3 are markedly upregulated (*p* < .05 or *p* < .01, Figure 8(A)). These genes are classical genes; for example, DDIT3 induces apoptosis, SQSTM1 is involved in autophagy, and genes such as FN1, CDH2, F2RL1, and MMP9 are responsive to metastasis. The classical genes also include those that govern the cell cycle, such as CDKN1B, CDK6, CDKN1A, and CDKN2A. Additionally, western blotting was used to validate the majority of key pathways, including the NF-κB, mTOR, and AKT pathways. The results indicated that p-AKT, p-mTOR, and p-NFκB-p65 were considerably downregulated (*p* < .05 or *p* < .01, Figure 8(B, C)). These findings demonstrated the upregulation of CDKN1B, CDKN1A, and DDIT3 and the downregulation of Slug, FN1, CDH2, F2RL1, CDK6, CCND1, and CDKN2A. According to this theory, all ten of these abnormally expressed genes are crucial for the ability of MKN-45 cells to migrate and stop their cell cycle after COPB2 suppression. Additionally, by selecting a pathway from our current study, more research is needed to elucidate the fundamental mechanisms of COPB2 in the evolution of GC.

RT‒qPCR and western blotting revealed that Slug, FN1, CDH2, F2RL1, CDK6, CCND1 MMP9, CDKN2A, and SQSTM1 are downregulated, and CDKN1B, CDKN1A, and DDIT3 are upregulated (*p* < .05 or *p* < .01; Figure 8A). In addition, western blotting revealed that the levels of the phosphorylated forms of p-AKT, p-mTOR, and p-NFκB-p65 were significantly lower (*p* < .05 or *p* < .01, Figure 8(B,C)). These results revealed that 12 aberrantly expressed genes play important roles in cell migration and cell cycle inhibition in MKN-45 cells via the AKT, mTOR, and NF-κB-p65 signaling pathways following COPB2 silencing. **p < .01, ***p < .001, compared with the Lv‑shCtrl group.

## Discussion

Although a previously published study revealed that inhibiting COPB2 might cause apoptosis and reduce cell proliferation in a variety of cancers, its function and importance in GC remain unclear. To understand the role of COPB2 in GC tumorigenesis, its functions, including cell migration, the cell cycle, and related mechanisms, need to be investigated. This research will hopefully lead to the discovery of a new, promising potential biomarker and target therapy gene. In this work, COPB2 silencing in MKN-45 cells markedly reduced the capacity of cells to migrate and stopped the cell cycle at the G0/G1 stage. After COPB2 was silenced, Affymetrix mRNA microarray analysis together with KEGG pathway enrichment analysis were carried out to screen pertinent molecules to investigate potential processes related to COPB2 silencing.

KEGG pathway enrichment bioinformatics revealed that a subset of genes with abnormal expression was associated with tumorigenesis-related pathways in GC. There is enough evidence to conclude that certain pathways, such as the NRF2-mediated oxidative stress response [14], the p53 signaling pathway [15,16], autophagy or regulation of autophagy [17], NF-kappa B signaling pathway [18,19], TNF signaling pathway [20], the FoxO signaling pathway [21], HIF-1 signaling pathway [22], and the TGF-beta signaling pathway [23], are involved in GC tumorigenesis. Furthermore, other pathways, including those involving proteoglycans in cancer, small cell lung cancer, bladder cancer, pancreatic cancer, non-small cell lung cancer, colorectal cancer, and glioma and pathways related to cancer, were also slightly linked to carcinogenesis. Consequently, there is clear evidence that COPB2 plays a role in the development of GC, which is responsible for the behavior of tumors, including the cell cycle, migration, and invasion. COPB2 also serves as an important GC biomarker for diagnosis and prognostic assessment.

After screening via the Affymetrix mRNA microarray assay, RT‒qPCR and western blotting validation revealed that Slug, FN1, CDH2, F2RL1, CDK6, CCND1 MMP9, CDKN2A and SQSTM1 are downregulated and that CDKN1B, CDKN1A and DDIT3 are upregulated. Slug [24,25], FN1 [26,27], CDH2 [28] and MMP9 [29,30] are classical molecules that are responsible for tumor metastasis, and their overexpression can promote tumor invasiveness and metastasis and even apoptosis. CDK6 [31], CCND1, CDKN2A, CDKN1B and CDKN1A are cell cycle markers that are responsible for controlling the G1 phase of the cell cycle. CDK6, CCND1, and CDKN2A are downregulated when the cell cycle is stopped at the G0/G1 phase, but CDKN1B and CDKN1A are upregulated. In addition, according to the diseases or functions of IPA, several genes are involved in apoptosis. A member of the protease-activated receptor family that binds to guanosine nucleotide-binding proteins is Factor II receptor-like 1 (F2RL1). It is overexpressed in multiple kinds of tumors and participates in tumorigenesis, such as breast cancer [32], hepatocellular carcinoma [33], colorectal carcinoma [34], ovarian clear cell carcinoma [35], pancreatic cancer [36], and prostate cancer [37]. F2RL1 is also overexpressed in GC, the overexpression of which can be easily detected in TCGA database-based online databases, such as GEPIA [13] and UALCAN [38]. DNA damage inducible transcript 3 (DDIT3) is an acronym for a protein that codes for an endoplasmic reticulum stress-activated protein that ultimately triggers apoptosis. Numerous studies have revealed that DDIT3 is downregulated in GC and that low DDIT3 expression is predictive of poor prognosis and is linked to clinical stage, tumor differentiation, and lymph node metastasis [39,40]. Sequestosome 1, also known as p62 or SQSTM1, is an acronym for a multifunctional protein implicated in gastric cancer autophagy [41,42]. SQSTM1 has been shown to be downregulated in GC, and low protein expression of SQSTM1 in gastric adenocarcinomas is positively correlated with poor prognostic factors such as survival and to be linked to the metastasis of GC to lymph nodes, vessels, and the liver [43]. As a result, SQSTM1 could be used as a prognostic biomarker and potential therapeutic target for gastric adenocarcinomas [43,44]. Many previous reports have demonstrated that p-AKT [45,46], p-mTOR [47,48], and p-NFκB-p65 [49,50] are involved in the regulation of cell proliferation, the cell cycle and cell migration. In the present study, western blotting was used to confirm the considerable downregulation of p-AKT/p-mTOR/p-NFκB-p65, which is responsible for the inactivation of the AKT, mTOR, and NF-κB signaling pathways.

Overall, COPB2 knockdown in GC cells significantly reduced the ability of cells to migrate and stopped the cell cycle at the G0/G1 stage. The mechanisms underlying these effects are linked to the upregulation of CDKN1B, CDKN1A, and SQSTM1 mediated by inactivation of the mTOR, AKT, and NF-κB signaling pathways and the downregulation of Slug, FN1, CDH2, F2RL1, CDK6, CCND1 MMP9, CDKN2A, and SQSTM1. Consequently, COPB2 May participate in migration and the cell cycle and could be a useful target for gene therapy approaches in the treatment of GC. In addition, much research is still needed to fully comprehend the role of COPB2 in the carcinogenesis of GC, including a number of pathways and aberrantly expressed mRNAs. The use of microarrays combined with IPA has undoubtedly contributed to the elucidation of the underlying mechanism and insight into the role of COPB2 in GC. The IPA contains 5 parts, including classic pathway analysis, disease and functional analysis, upstream regulation analysis, regulatory effect analysis, and interaction network analysis. In this study, only classic pathway, disease and functional analyses were explored, and all other methods were fully validated and developed.

While our earlier work pinpointed specific stress/apoptosis and RTK signaling nodes, the present multiomics approach reveals a central role for the PI3K/AKT/mTOR and NF-κB cascades. These findings suggest that COPB2 May act as a master regulator upstream of multiple critical oncogenic pathways. The downregulation of p-AKT and p-mTOR provides a more integrated explanation for the observed anti-proliferative and pro-apoptotic effects, potentially linking the disparate pathways identified in our prior studies. A key advancement of this study is the demonstration that COPB2 silencing impedes GC metastasis, as evidenced by reduced migration and invasion in vitro. This functional aspect was not addressed in our previous reports and significantly broadens the potential therapeutic implications of targeting COPB2, as it could impact both tumor growth and dissemination. Unlike our prior candidate-based approaches, the utilization of an unbiased mRNA microarray and Ingenuity Pathway Analysis allowed for a systems-level investigation. This led to the identification of 12 critical genes (e.g., Slug, FN1, and CDKN1B) involved in EMT and cell cycle regulation, providing a more comprehensive understanding of the molecular landscape underlying the function of COPB2.

Despite the strengths of our study, several limitations should be acknowledged. First, our findings are derived primarily from retrospective bioinformatic analyses. The biological functions and mechanistic roles of the identified genes warrant further investigation through in vitro and in vivo experimental models, such as patient-derived xenografts or bioengineered human disease models, to elucidate the underlying biology. Second, the clinical correlation and translational potential of our signature, while promising, require validation in prospective, multicenter trials to assess its real-world utility and incremental value over standard clinicopathological parameters.

## Data Availability

The datasets generated and analyzed during the present study are available from the corresponding author upon reasonable request.

## Abbreviations

TMA: Tissue Microarray
IPA: Ingenuity Pathway Analysis
KEGG: Kyoto Encyclopedia of Genes and Genomes
GC: Gastric Cancer
TCGA: The Cancer Genome Atlas
RT‒Qpcr: Quantitative Real-time PCR
PBS: phosphate-buffered saline
DEGs: differentially expressed genes
